# Factors affecting the use of long term and permanent contraceptive methods: a Facebook-focused cross-sectional study

**DOI:** 10.1186/s12905-022-01784-0

**Published:** 2022-06-02

**Authors:** Naser Al-Husban, Dalia Kaadan, Jude Foudeh, Tara Ghazi, Yumen Sijari, Maher Maaita

**Affiliations:** 1 School of Medicine, The University of Jordan, P O Box 2194, Amman, 11941 Jordan; 2 Obstetrics and Gynecology Department, The Royal Medical Services, Amman, Jordan

**Keywords:** Contraception, Sterilization, IUCD, Vasectomy, Tubal, Ligation

## Abstract

**Introduction:**

In the Muslim world, the use and acceptance of long-term and permanent contraceptives were limited. Our aim was to investigate those limiting factors so we can help making these methods widely available and acceptable to the society.

**Methods and data analysis:**

There were 1365 women from Facebook groups in the period 08/10/2020–8/11/2020. Participants were married women, living in Jordan. This was a cross-sectional study. Statistical Package for Social Sciences (SPSS), version 16, software was used for statistical analysis (Chicago, Illinois, USA).

**Results:**

Among participants, 22.3% had never used any contraceptives. Non-hormonal IUCD was the most commonly used method. There was a statistically significant association between the use of hormonal IUCD and women's age, marriage duration, education and number of children (*p* < 0.0001). Tubal ligation was adopted by only 44 (3.22%) participants. 19.68% of participants declined tubal ligation merely due to religious issues. Women who completed only high school level of education underwent tubal ligation significantly more than those with university (Bachelor) and post-university (Master or PhD) degrees (*p* < 0.0001 and 0.026, respectively). Only 1.83% of women's partners underwent vasectomy, the majority of these vasectomies (72.0%) were done because of the need for lifelong contraception. Around 17% of women's partners had poor knowledge about vasectomy. Further, women's employment status (housewives or full-time employees) was found to be the only variable that affected acceptance of vasectomy (*p* = 0.0047).

**Conclusions:**

Women endured a heavy burden of contraception. Cultural and religious taboos influenced tubal ligation. Vasectomy was still very rarely adopted by men due to the lack of knowledge about the procedure. Our results raised the need for further dissemination of contraception knowledge and counselling through the primary care and maternity centers, mosques and media in official, comprehensive and integrated programs. Future research is needed in the field of permanent contraceptive methods.

## Introduction

There have been huge developments in contraceptive methods [1]. The most commonly known methods among women in Jordan were IUD followed by the pill and withdrawal methods which all together represent (94%) [2].

Contraceptive practices have increased after the approval of contraception by Islamic rulings [3]. Contraceptive use was found to be associated with markers of socioeconomic status of women in Jordan [3]. On top of these markers was women’s education. Therefore, it has become imperative to strengthen school health programs with regard to family planning and increase the age of getting married to empower young girls to make decisions on the future of their reproductive health [3, 4].

Religious scholars argue that family planning as an external western conspiracy aiming at curtailing the growth and strength of the Islamic world [4]. These arguments appear to be uninformed of both the socio-political and demographic realities in many Muslim countries [5]. In other words, profound socio-economic and political difficulties in different Muslim countries in addition to Jordan such as the lack of family planning and population explosion would slow down and stifle the process of overall development [4–6].

### Rationale, scope and aim of our study

There is a lack of research among Jordanian women about the use of long-acting and permanent contraceptive methods and the factors that may affect their uptake. In addition, there are religious, socio-economic and political difficulties in the country with the ongoing unplanned pregnancy that not only fuels social, financial and family difficulties but also threatens some women's lives, especially, those who suffer from certain health problems. The uptake of permanent contraceptives, namely tubal ligation and vasectomy in Jordan is very limited. Therefore, this study aimed to explore the factors influencing the use of long-acting and permanent contraceptive methods so that health policy makers can find practical solutions to make these methods, particularly tubal ligation and vasectomy, widely available and acceptable to the society. This will allow both women and men to have more options regarding their reproductive health.

## Materials and methods

We performed a cross-sectional study utilizing a “validated” questionnaire that was disseminated using Facebook groups in Jordan. We posted the survey for several personal pages and social public pages. It was really hard to assess the reach of this survey. However, this was a convenient sampling methodology in which people who were interested had filled in the survey without knowing the percentage of response. We managed to include women groups from different areas of Jordan via Facebook as the vast majority of Jordanians were Facebook users. It was during the time of the COVID-19 pandemic with significant restrictions and curfews across the country. In addition, there were many Jordanian women groups on Facebook. Therefore, data were collected from participants using Facebook groups only. The use and acceptance of long-term and permanent contraceptives were studied in relation to women's age, level of education, place of residence, duration of marriage, number of children, women's employment status and family's monthly income.

Participants were women in the reproductive age group living in Jordan who received the questionnaire and agreed to complete it. The questionnaire was available online to women for around one month (08/10/2020–8/11/2020).

Women who were healthy, aged 16–49, married, non-pregnant at the time of filling the questionnaire and have been living in Jordan for the last 10 years were included. The 10-year-residency period was chosen as copper IUCD might be used for 10 years. Moreover, those who were living abroad might have their contraceptive choices been influenced by method availability, culture and insurance issues in the country that they were living in. Therefore, we could avoid the bias caused by foreign countries' culture and contraceptive practices.

Excluded candidates were divorced or non-married women, those who were pregnant at the time of the study and Jordanian women living abroad. Divorced and non-married women were excluded as it is very critical for these women to explore their sexual practices due to religious, social and cultural issues even after being reassured regarding strict confidentiality.

The questionnaire was distributed to Jordanian women Facebook groups because of the COVID-19 pandemic, and maximum efforts were exerted to ensure that all areas in Jordan were covered. Upon obtaining permission from the authors [7] of the original questionnaire, it was translated into Arabic and customized to the culture of the Jordanian community. The questionnaire contained a total of 57 questions divided into 5 sections (see Appendix 1). This appendix illustrated how the variables were collected and categorized. The first section included a description about the study explaining its importance, then moving to the second section into which consent was needed to be checked in order to continue into the next section. The third section consisted of 8 questions which assessed the sociodemographic characteristics of the participants. The fourth section included 27 questions which assessed the level of knowledge of participants regarding LAPM’s. Long-acting contraceptives were defined as those lasting 3 or more years and being reversible. These included hormonal and non-hormonal IUCDs. Permanent methods were defined as those that were considered irreversible and everlasting. They included tubal ligation and vasectomy. The fifth and last section was comprised of 21 questions which tackled the use of long-term contraceptive methods and their side effects on Jordanian women. The questions were related to past and current contraception experience. The questionnaire was first validated by distributing it to 32 Jordanian women of different social, financial and level of education backgrounds. They completed the questionnaire and confirmed its suitability to the Jordanian society. The data were obtained via the questionnaire which was completed voluntarily by the women in question. To reduce any potential bias in the result, the questionnaire was distributed to all accessible women living across the country.

Sample size was calculated to be 1300 subjects with a 90% power and 0.05 alpha.

Internet services in Jordan were widely accessible and the vast majority of Jordanians were Facebook users, therefore, we thought that our cross-sectional study could be a reasonable representation.

### Statistical analysis

After data collection, an excel spread sheet was generated and the data was analyzed using SPSS version 24. The analysis of the data employed a descriptive statistical approach to analyze the study’s population in relation to the relevant variables. Both univariate and bivariate analysis were carried out. To ensure the significance of the result, two statistical tests were used; namely, Chi-square and likelihood ratio with 95% confidence interval were employed as appropriate. *P* value < 0.05 was taken as a cut point for significance. For Table 2, we used two-by-two table with the relative risk and odds ratio to show the exposure and non-exposure to the contraceptive and for the relationship between two binary quantities. The relative risk analysis to get the odds ratio was to measure the association of two groups using the Pearson chi square and Fisher exact test if the cell was < 5. The dependent variable was the cause and the independent was the effect. In our study, the contraceptive was changing as per the education level, age and duration of marriage while the variables' probability was checked by linear correlation.

We assessed the association between these variables (independent and dependent) using binary analysis and then we performed a logistic regression analysis and quantify this association. The comparison was made to determine the differences attributed to the respective contraceptive; however, the characteristics varied in the population. The results showed the likelihood that there was a difference between the types of the used contraceptives.

### Ethical consideration

Ethical clearance and permission were obtained from the Institutional Review Board (IRB) of the Jordan University hospital, approval number 278/2020 dated 17/11/2020. Consent and privacy of participants were ensured at the beginning of the questionnaire. The data was completed anonymously and confidentiality was maintained at all times. It was registered in Clinicaltrials.gov; (ID; NCT04672304).

Informed consent was obtained from all the participants.

All methods were performed in accordance with the relevant guidelines and regulations.

## Results

The questionnaire was received by 5000 women. The response rate was 44.8% (*n* = 2240). The respondents, 2240 women were seen as a fair response regarding contraceptive use in a country with a total population of 10 million people. Eight hundred women were excluded and 75 responses had missing data. Finally, 1365 women were included in this study. Overall, 77.7% had used at least one contraceptive method during their lifetime, and 22.3% had never used any contraceptives. About 47.6% of them were in the age group (31–40) years old. About 55% of the participants were unemployed. The majority was living in the middle territory (82.9%). It was found that 44.2% of the participants had monthly income > 1000.0 Jordan Dinars (JDs) (equivalent to 1316.0 USD), and about 17.9% had an income < 500.0 JDs (658.0 USD) (see Table 1).

**Table 1:** Detailed demonstration of socio-demographic characteristics of the women and their relation to the use of long acting and permanent contraceptive methods with distribution of women among different categories

	N	%	Hormonal IUCD		Non-hormonal IUCD		Tubal ligation		Vasectomy		Total contraceptive users		Total non- contraceptive users	
			N	%	N	%	N	%	N	%	N	%	N	%
*Age group*														
16–25	101	7.4	5	3	16	2.8	2	4.5	3	12	26	3.2	75	13.4
26–30	302	22.1	23	13.6	73	12.9	8	18.2	10	40	114	14.2	188	33.5
31–40	650	47.6	75	44.4	296	52.3	18	40.9	9	36	398	49.5	252	44.9
41–50	312	22.9	66	39	181	32	16	36.4	3	12	266	33.1	46	8.2
Total	1365	100	169	100	566	100	44	100	25	100	804	100	561	100
P-Value			< 0.001		< 0.001		0.1809		0.092					
*Duration of marriage*														
1–10	731	53.6	49	29	195	34.5	16	36.4	19	76	279	34.7	452	80.6
11–20	442	32.4	72	42.6	235	41.5	14	31.8	4	16	325	40.4	117	20.9
21–30	165	12.1	41	24.3	116	20.5	12	27.3	2	8	171	21.3	-6	-1.1
31–40	27	2	7	4.1	20	3.5	2	4.5	0	0	29	3.6	-2	-0.4
Total	1365	100	169	100	566	100	44	100	25	100	804	100	561	100
P-Value			< 0.001		< 0.001		0.0053		0.1471					
*Level of education*														
University (Bachelor)	871	63.8	83	49.1	340	60.1	21	47.7	14	56	458	57	413	73.6
College (Diploma)	149	10.9	17	10.1	77	13.6	5	11.4	4	16	103	12.8	46	8.2
Post-university (Master and PhD)	196	14.4	39	23.1	74	13.1	6	13.6	6	24	125	15.5	71	12.7
High School graduate	142	10.4	29	17.2	71	12.6	12	27.3	1	4	113	14.1	29	5.2
Middle School	7	0.5	1	0.6	4	0.7	0	0	0	0	5	0.6	2	0.4
Total	1365	100	169	100	566	100	44	100	25	100	804	100	561	100
P-value			0.001		0.0124		0.0104		0.6031					
*Residency*														
Middle (central)	1130	82.8	131	77.5	437	77.2	29	65.9	17	68	614	76.4	516	92
North	184	13.5	28	16.6	95	16.8	13	29.5	6	24	142	17.7	42	7.5
South	51	3.7	10	5.9	34	6	2	4.5	2	8	48	6	3	0.5
Total	1365	100	169	100	566	100	44	100	25	100	804	100	561	100
P-Value			0.6222		0.0109		0.4437		0.3744					
*Number of Children*														
0	110	8.1	3	1.8	6	1.1	4	9.1	5	20	18	2.2	92	16.4
1–3	917	67.2	88	52.1	322	56.9	14	31.8	14	56	438	54.5	479	85.4
More than 3	338	24.8	78	46.2	238	42	26	59.1	6	24	348	43.3	-10	-1.8
Total	1365	100	169	100	566	100	44	100	25	100	804	100	561	100
P-Value			< 0.001		< 0.001		< 0.001		0.0827					
*Employment*														
Housewife (unemployed)	751	55	99	58.6	326	57.6	28	63.6	7	28	460	57.2	291	51.9
Student	4	0.3	1	0.6	2	0.4	0	0	0	0	3	0.4	1	0.2
Part-time Employee	145	10.6	17	10.1	61	10.8	2	4.5	2	8	82	10.2	63	11.2
Full-time Employee	458	33.6	52	30.8	175	30.9	13	29.5	15	60	255	31.7	203	36.2
Other	7	0.5	0	0.0	2	0.4	1	2.3	1	4.0	4	0.5	3	0.5
Total	1365	100.0	169	100.0	566	100.0	44	100.0	25	100.0	804	100.0	561	100.0
P- Value			0.6482		0.4744		0.2587		0.0047					
*Monthly income*														
< $ 658.0	245	17.9	33	19.5	92	16.3	9	20.5	6	24.0	140	17.4	105	18.7
$ 658.0–1316.0	516	37.8	56	33.1	227	40.1	17	38.6	9	36.0	309	38.4	207	36.9
> $ 1316.0	604	44.2	80	47.3	247	43.6	18	40.9	10	40.0	355	44.2	249	44.4
Total	1365	100	169	100	566	100	44	100	25	100	804	100	561	100
P- Value			0.3840		0.0723		0.8685		0.7243					

It also shows the number and percentage of contraceptive users and non-users in these categories

*N* number, *IUCD* intra-uterine contraceptive device

### Source of information about contraceptive methods and the participants’ choice of contraception

Contraceptive knowledge was obtained from healthcare personnel at hospitals in 74.8% of participants, 39.5% from the Internet and social media and 26.1% of participants got that knowledge from their family members and relatives.

47.6% of the participants claimed that gynaecologists had high impact on their choice of the contraceptive method. On the other hand, 31.7% said that their effect was moderate, and 19.8% were minimally affected by their doctor’s opinion.

### Variables that affected the use of non-hormonal intra-uterine contraceptive device (IUCD)

Non-hormonal IUCD was found to be the most commonly used method among married Jordanian women in our study. It was used by 41.5% of the participants at least once in their life time; 61.08% used it for less than 5 years, 32.15% used it for 5–10 years, and 6.77% used it for more than 10 years. The most common reasons for choosing this method over other contraceptive methods were attributed to the following reasons. 59.36% of the participants expressed that this method did not need compliance, 58.30% stated it prevented pregnancy for a long period of time, and 55.84% conveyed it could be reversed. On the other hand, the most common causes for discontinuing the use of non-hormonal IUCD were (1); 91.17% of the participants expressed they discontinue this method for the desire to have more kids, (2); 69.96% they stop it because it could led to heavy menstrual bleeding that it could cause, and (3); 57.24% mentioned due to reaching the maximum duration of usage.

The *p* value was found to be significant regarding the correlation between the use of non-hormonal IUCD with the age the participant, duration of marriage, level of education, the number of children, and residency area at *p* < 0.001, *p* < 0.001, *p* = 0.0124, *p* < 0.001 and *p* = 0.0109, respectively. It was not statistically and significantly different in relation to job (employment) or monthly income *p* = 0.5 and *p* = 0.07 respectively (see Table 1).

Further analysis showed that, those who were older than 40 years old were using it more than those in the 16–25, and 26–30 age groups (p < 0.001). Those who have been married for 11–20 years were also using it more than other groups (p < 0.001). It was also more significantly used by university (Bachelor) and post-university (Master and PhD) qualified women than other educational categories (p < 0.001 and *p* = 0.025) (see Table 2).

**Table 2:** Influence of different variables on the various contraceptive methods

	Age group	N	OR	(CI 95%)		P-Value
*Age group*						
Hormonal IUCD N = 169						
> 40 years N = 66	16–25	5	0.1947	0.0674	0.4678	**< 0.00**1
	26–30	23	0.3078	0.1829	0.5055	**< 0.00**1
	31–40	75	0.4866	0.3382	0.700	**< 0.001**
Non-Hormonal IUCD N = 566						
> 40 years N = 181	16–25	16	0.2608	0.1422	0.4585	**< 0.00**1
	26–30	73	0.441	0.311	0.622	**< 0.00**1
	31–40	296	1.155	0.8796	1.5.19	0.3011
Tubal ligation N = 44						
> 40 years N = 296	16–25	2	0.3744	0.0573	1.45	0.1782
	26–30	8	0.1131	0.5039	0.2012	1.185
	31–40	18	0.0636	0.5273	0.2631	1.064
Vasectomy N = 25						
> 40 years N = 3	16–25	**3**	3.142	0.5323	18.55	0.1426
	26–30	**10**	3.521	1.009	16.04	**0.0432**
	31–40	**9**	0.580	1.446	0.4053	6.661

	Duration of marriage	N	OR	(CI 95%)		P-Value
*Duration of marriage*						
Hormonal IUCD N = 169						
1–10 N = 49	11–20	72	2.706	1.845	3.993	**< 0.001**
	21–30	41	0.591	2.897	7.26	**< 0.001**
	31–40	7	4.853	1.83	11.8	**< 0.00**1
11–20 N = 72	21–30	41	0.5891	0.3822	0.9143	**0.016**
Non- Hormonal IUCD N = 790						
1–10 N = 195	11–20	235	2.071	1.604	2.677	**< 0.00**1
	21–30	116	4.313	2.969	6.324	**< 0.001**
	31–40	20	5.201	2.212	13.43	**< 0.001**
11–20 N = 235	21–30	116	0.4801	0.3256	0.7019	**< 0.001**
	31–40	20	2.512	1.065	6.501	**0.034**
Tubal ligation N- 44						
1–10 N = 16	11–20	14	0.6843	0.3277	1.442	0.3054
	21–30	12	0.2859	0.1318	0.6323	< 0.001
	31–40	2	0.2806	0.069	1.891	0.2634
21–30 N = 12	11–20	14	0.4178	0.187	0.944	0.0263

	Level of education	N	OR	(CI 95%)		P-Value
*Level of education*						
Hormonal IUCD N = 169						
Post University N = 39	University(Bachelor)	83	2.359	1.543	3.57	**< 0.001**
	College (Diploma)	132	1.925	1.049	3.635	**0.034**
	High School	29	0.9424	0.5495	1.627	0.829
High School N = 29	University (Bachelor)	83	0.4025	0.2536	0.6497	**< 0.00**1
	College (Diploma)	132	0.4897	0.2511	0.9352	**0.029**
Non- Hormonal IUCD N = 566						
Post University N = 74	University (Bachelor)	340	1.056	0.768	1.457	0.7396
	College (Diploma)	77	1.760	1.143	2.719	**< 0.00**1
	High School	71	1.646	1.062	2.557	**0.025**
University N = 340	High school	71	1.561	1.092	2.232	**0.014**
	College (Diploma)	77	1.669	1.177	2.37	**0.004**
Tubal Libation N = 44						
High School N = 12	University (Bachelor)	21	3.817	1.78	7.916	**< 0.001**
	College (Diploma)	5	2.712	0.948	8.77	0.058
	Post University (Master, PhD)	6	0.982	1.1	8.809	**0.026**

	No. of children	N	OR	(CI 95%)		P-Value
*No. of children*						
Hormonal IUCD N = 169						
≥ 4 N = 78	0	3	10.67	3.395	53.99	**< 0.001**
	1–3	88	2.993	2.13	4.205	**< 0.001**
Non- Hormonal IUCD N = 566						
≥ 4 N = 238	0	6	0.025	0.009	0.054	**< 0.00**1
	1–3	222	0.1571	0.118	0.207	**< 0.001**
Tubal ligation N = 44						
≥ 4 N = 26	0	4	0.4535	0.1325	1.244	0.1992
	1–3	14	0.1864	0.09362	0.3592	**< 0.00**1

	Residency	N	OR	(CI 95%)		P-Value
*Residency*						
Hormonal IUCD N = 169						
Central N = 131	North	28	1.368	0.868	2.11	0.162
	South	41	1.859	0.8669	3.711	0.084
Non- Hormonal IUCD N = 566						
Central N = 437	North	95	0.591	0.432	0.809	**< 0.001**
	South	34	0.3156	0.171	0.568	**< 0.001**
Tubal Ligation N = 544						
Central N = 29	North	13	2.883	1.426	5.6	**0.001**
	South	2	1.549	0.243	5.747	0.782

Significant *P* values are bolded

*N* number, *IUCD* intra-uterine contraceptive device, *Sig.* significant, *CI* confidence interval, *OR* odds ratio

Furthermore, non-hormonal IUCD was used by those who had 4 or more children and more than those who had fewer children, this was statistically significant, *p* < 0.001 (see Table 2). Those who were resident in the central region (mainly the capital) were more statistically and significantly used it more than those who were living in either north or south regions *p* < 0.001 and < 0.001, respectively (see Table 2).

### Variables that affected the use of hormonal IUCD among participants

Hormonal IUCD was used by 15.93% of the participants. The most commonly reported reasons for the use of this method were; high efficacy 49.39%, long-acting 45.12% and its reversibility 32.32%.

There was a statistically significant association between the use of hormonal IUCD and the following variables: age, duration of marriage, level of education and number of children *p* =  < 0.001, *p* < 0.001, *p* < 0.001, *p* < 0.001 respectively. Its use was not affected by job (employment), residency or monthly income *p* = 0.6, *p* = 0.6 and *p* = 0.4, respectively (see Table 1).

Further sub-analysis of these factors that affected the hormonal IUCD use showed that women who were more than 40 years old statistically and significantly used it more than all other age groups *p* < 0.001. Those with a duration of marriage between 1–10 years used it more than all other duration of marriage groups; a statistically significant difference *p* < 0.001. Moreover, those with 11–20 years of marriage used it more than those with 21–30 years, again a statistically significant difference *p* = 0.016. We also found that those with post-university (Master and PhD degrees) education and those with only high school level used the hormonal IUCD more than those with university (bachelor) or College (diploma) qualification *p* < 0.001, *p* = 0.034, *p* < 0.001, *p* = 0.029 respectively. Also, those who had 4 or more children used this method more significantly than those with fewer children *p* < 0.001 (see Table 2).

### Variables that affected the use of Tubal Ligation and vasectomy among participants

There were 4.15% of women who underwent tubal ligation as a method of contraception. Of those, 65.81% had chosen this method due to lifelong contraceptive effect of this procedure, while 56.81% preferred tubal ligation because they have no desire to have more kids. Tubal ligation was not an option for the majority of Jordanian women due to the wish to conceive in 21.65% and religious causes in 19.68%. It was found that 21.65% of women did not opt for tubal legation as they wish to conceive whereas19.68% did not use this method due to religious causes.

There was a significant correlation between undergoing tubal ligation and the duration of marriage, level of education, and number of children the women had *p* = 0.0053, *p* = 0.0104 and *p* < 0.001, respectively (see Table 1).

Tubal ligation was seen more in women with duration of marriage of 21–30 years than those with either 1–10 or 11–20 years, this was a statistically significant finding *p* < 0.001 and *p* = 0.026 respectively. Women with high school level of education underwent tubal ligation significantly more than those with university (Bachelor) or post-university (master or PhD) levels between *p* < 0.001 and *p* = 0.026). Women with 4 or more children who had a tubal ligation were significantly more than those who had fewer children *p* < 0.001 (see Table 2). More women living in the central region of the kingdom underwent tubal ligation than those living in the north, *p* < 0.001 but not significantly different in relation to those living in the south, (see Table 2).

1.83% of women in the study reported that their spouses underwent vasectomy. The reasons behind choosing this method were mostly due to the need for lifelong contraception in 72.0%. Not choosing this method was mostly attributed to the wish to have more children 20.15% and having poor knowledge regarding this procedure (17.01%). Women who were full-time employees and housewives had a significantly more spouses who underwent vasectomy more than other categories (*p* = 0.0047) Table 1. Moreover, women older than 40 years had a slightly higher chance to have spouses who underwent vasectomy, *p* = 0.0432, Table 2.

### Effects of employment (job) and residency

Types of employments didn't have any statistical significance in terms of using non-hormonal IUCD, *p* = 0.5, the hormonal IUCD, *p* = 0.6 and tubal ligation, *p* = 0.3, (see Table 1).

It was found that the place of residency pf women did not have any statistical significance on whether women, in Jordan, use Hormonal IUCD or vasectomy *p* = 0.6, and *p* = 0.4, respectively (see Table 1).

### The effect of monthly income: (< $ 658.0, $ 658–1316.0, and > $ 1316.0)

Monthly income did not significantly affect hormonal IUCD use, (*p* = 0.4), non-hormonal IUCD, *p* = 0.07, tubal ligation *p* = 0.9, and vasectomy *p* = 0.7 (see Table 1).

### General knowledge about long acting and permanent contraceptive methods

Participants who answered 70% of the questions correctly were considered to be well educated about the aforementioned methods. Out of the 56.3% of the participants who used non-hormonal IUCD method, 43.8% had good knowledge about it. On the other hand, the general knowledge about the hormonal IUCD was found to be low. It was found that only 8.6% had good knowledge about it (see Table 3).

**Table 3:** The relationship between the knowledge of the participants about the different contraception methods and the use of each of those methods

Method	Non-hormonal IUCD	Hormonal IUCD	Tubal ligation	Vasectomy
*Knowledge*				
Poor	28.55%	91.4%	3.08%	1.90%
Good	56.35%	8.6%	3.35%	1.30%
*P* value	< 0.0001	< 0.0001	0.3537	0.6081

*IUCD* intra-uterine contraceptive device

52.4% of the participants had high level of knowledge about tubal ligation; however, only 3.4% underwent this procedure. While 11.2% had good knowledge about vasectomy, only 1.3% of those who had good knowledge about it had actually used this method (Table 3).

## Discussion

### Findings and interpretation

Delayed age of marriage in Jordan was reflected on our study as there were only 7.4% of women in the age group 16–25 years and the majority of those in the age group of 31–41 hold a university bachelor degree. Because of cultural and religious constraints, there is practically no sex before marriage in Jordan and, consequently no need for teenage contraception. The minimum age for marriage in Jordan was 18 years [6]. Jordan’s parliament had voted to make the minimum age of marriage in “exceptional” cases from 15 to 16 years to reduce child marriage rates in the country. In exceptional circumstances, a judge can approve marriage to a minor if a sharia committee deems it necessary and with both parties' consent [6]. Slightly over half of the women in this study were not employed. Employment rate in Jordan decreased to 26.20% in the third quarter of 2020 [8]. The monthly income in Jordan stands at around $637.0 [8].

The majority of the population is living in the middle region. This region has financial, medical, educational and employment advantages over the north and south regions. However, it is more culturally open and less conservative than the other regions. People living in the northern region are more involved in agriculture due to the availability of water and fertile lands. They usually need big families and this might have contributed to significantly less tubal ligation in this region [8].

The type of non-hormonal IUCD in Jordan was the Copper IUCD (TCu380A) which has duration of continuous use up to 10 years [9]. The commonest reasons for this choice in our study were that it was user-independent, its long-term efficacy and reversibility. However, the commonest reason for its removal was a desire to have more kids. The insertion and removal of the non-hormonal IUCD device in Jordan were offered free of charge in the maternal and child centers across the country. This scheme was funded by the government and the USAID. Therefore, its use was not affected by employment status or monthly income. We did not study the effect of husbands' views and support on the use or discontinuation of non-hormonal IUCD in our population. However, the use of copper IUCD was found to be related to the support of husbands, knowledge about its safety, efficacy, and counseling [10]. Our study showed that its use was strongly associated with women's age being above 40 years, duration of marriage, level of education, number of children (4 or more) and being resident in the central region. This method was convenient for the participants in this subgroup of women who were supposed to be busy and engaged in so many life issues. In support of this finding, in nulliparous women, IUCD continuation rate after one-year follow-up was 90.3%, satisfaction rate was high 93.8% and patients tolerance was also good [11]. Menstrual amount was found to be increased for 84%, which was consistent with the findings of our study [11].

Contrary to the non-hormonal IUCD, the hormonal IUCD is expensive in Jordan (the device costs around 142.0 USD) and is not covered by any insurance. Its insertion and removal also incur further cost. Therefore, it was used by only 15.93% of the overall women in our study. The reasons for the use and the discontinuation were similar to those for the non-hormonal IUCD. One study found that out of the 370 women who were counselled in the immediate postpartum period, 35.67% accepted the non-hormonal IUCD [12].

In our study, one of the reasons for the discontinuation of the hormonal IUCD device was the development of irregular menses in 28.75%. Abnormal bleeding after 3 months of use was found in 42% of cases in another study [13]. Around one third of users kept it for the whole recommended duration of use [13]. This could be due to the beneficial effects in controlling heavy menstrual bleeding as well as its contraceptive efficacy [13–16]. Moreover, 22.9% of our study population was between 40–50 years old. This was the age when problems of heavy bleeding, menorrhagia, increased benign lesions of the uterus were found to be increased. It was found in a prospective observational study that hormonal IUCD was a safe and effective option for menorrhagia in peri-menopausal women [17]. These beneficial features could be the reason behind the fact that its use was not affected by factors such as different types of jobs, area of residency or monthly income in our study.

Moreover, these features could explain the statistically significant difference of using hormonal IUCD by those who were more than 40 years old. In our study, the beneficial effects could also explain why women with only high school level or post-university qualification used it more than those with diploma or university level. The same explanation was applicable to those who had 4 or more children. In our study, hormonal IUCD was the second commonest contraceptive used method. Globally, hormonal IUCD was the second most commonly used form of reversible contraception due to its safety, high efficacy, convenience and cost effectiveness [17, 18].

Our study observed a statistically significant association between tubal ligation and duration of marriage of 21–30 years and with those living in the urban cities and central region. This was most likely due to the easy and direct access to family planning services and to surgical gynecologists that were capable of doing laparoscopic procedures. One study was found that women from rural counties were more likely to undergo a tubal ligation than their urban counterparts [19]. On the contrary, the shortage of trained providers, particularly in rural areas, was a critical barrier for accessing high-quality family planning equitably [20].

Moreover, the Jordanian law was changed to demand the approval of two consultant obstetricians/gynaecologists for any tubal ligation. Our study also showed that adopting tubal ligation in Jordan was still significantly influenced in a negative way by false religious beliefs. The vast majority of the Jordanian people are Sunni Muslims with a Christian minority. Irrespective of their faiths, Jordanians are very conservative and religious. In fact, the true Islam does not forbid or regard tubal ligation as a sin. To increase tubal ligation adoption in Jordan, more work in terms of counselling and education about true Islamic views are needed as other faiths constitute only a minority. The majority of the obstetricians and gynaecologists in Jordan don’t share these views and are happy to perform tubal ligation. Similarly, a USA study found that obstetricians/gynecologists working in Catholic hospitals did not share the Church’s belief on sterilization [21].

In our study, tubal ligation was seen significantly among those with only high school level of education, with 4 or more children and by those with 21–30 years of marriage. These associations could easily be explained as these women got married at an early age, had kids early and had a stable marriage relationship.

Extended families are widely prevalent in Jordan [22]. Moreover, families usually gather together and are very social with female relatives having major influence on other women's opinion and attitudes regarding reproductive life [22]. We should make use of this social advantage to improve the uptake of tubal ligation in Jordan, taking into consideration the difficult economic situation both inside Jordan and across the globe. The role of mothers in contraception decision-making should be considered in designing and implementing family planning service [23].

### Similarities in relation to other studies

Only 1.83% of women's husbands in our study had vasectomy and this was significantly evident especially among women who had full-time employment. Poor knowledge regarding this procedure was found in 17.01%. These findings raised the issue that contraception in Jordan heavily relied on women and that there was a serious poor knowledge about this procedure. Women were unequivocally shouldering the main responsibility of contraception [24]. Male contraceptive methods accounted for only 14% of those used worldwide [25]. Vasectomy was utilized by 6–13% of American couples [26]. In Jordan, we should focus on involving male partner in counselling. We did not explore the details behind the poor knowledge of this method in our population. A study found that the level of knowledge about vasectomy was poor and educational status was an important predictor of that knowledge [27, 28]. Common questions about vasectomy should be answered as being a safe, effective, and permanent method with an overall failure rate of less than 1% in pooled studies [29]. Furthermore, reassurance regarding local anesthesia for vasectomy should be provided [30].

### Differences in our study

An alarming 22.3% of the women in our study never used a contraceptive in their lives. This raised issues of culture, religion, economy, access to medical care and the role of primary care centers and maternity units. Moreover, 26.1% of the participants obtained their information from non-professional personnel, namely, their family members and relatives. This raised the need for further dissemination of knowledge and counselling through the primary care and maternity centers in official, comprehensive and integrated programs with adequate time being given to women. These programs also are expected to increase the role of gynecologist on the uptake of the contraceptive advice. This role was shown in our study to be minimal or moderate between 19.8% and 31.7%.

### Strengths of the study

This study investigated several variables affecting the use of contraceptives including age, level of education, employment status, family size and different areas of residency within the kingdom which solidified the study. It incorporated participants with different age, social, economic, educational and residency backgrounds. Our study results might be generalizable to many Arab and Islamic countries. Our findings were relevant to the implications for clinicians and policy-makers/health care providers and future research.

### Limitations of the study

The slightly restricted pattern of distribution of the questionnaire due to the Covid-19 pandemic and low response rate were weak features of this study. Nevertheless, that was the most feasible sampling methodology at the time of COVID crisis. Moreover, the reach of Facebook users was easier to reach our target sample size. Moreover, our study did not investigate the influence of mothers and partners on women's contraceptive choice. Having said that, further studies should be conducted involving community, health and religious members.

### Unanswered questions and future research

Further work is required to promote the permanent contraception methods especially vasectomy due to its efficacy and low cost. This needs increasing cultural awareness, religious explanation, and education. We should take into account research exploring the health benefits of family planning on both maternal, child, and family health and prosperity.

Male contribution to contraception, especially when pregnancy threatens and endangers the women's lives, should be explored further as some women carry high surgical and anesthetic risks even when it comes to laparoscopic tubal ligation. This can be encouraged in Jordan and in the Muslim world as there are no clear religious objections to male contraception in Islam. Men should be aware that vasectomy is not forbidden in the true Islam, particularly when the pregnancy or the tubal ligation itself poses a threat to their wives' health or life.

## Conclusions

Jordanian women carry the heavy burden of contraception. Tubal ligation was still hampered due to cultural and mainly religious taboos and it was mainly utilized by women living in big cities. On the other hand, vasectomy in Jordan was still very rarely adopted with wide poor knowledge. These findings raised the need for further dissemination of knowledge and counselling through the primary care and maternity centers in official, comprehensive and integrated programs. Further research in Muslim communities with a focus on the need for proper advice regarding permanent contraceptives is recommended.

## Data Availability

The datasets generated and/or analyzed during the current study are not publicly available due to sensitivity issues with contraception in Jordan and are available from the corresponding author on reasonable request.

## Abbreviations

JPFHS: Jordanian Population and Fertility Health Survey
TFR: Total fertility rate
IUD: Intra-uterine device
OC: Oral contraceptive
LAPM: Long- acting and permanent contraceptive methods
IRB: Institutional Review Board
$: United States dollar
IUCD: Intrauterine contraceptive device
USAID: The United States Agency for International Development
N: Number
OR: Odds ratio
CI: Confidence interval
